# NSAIDs utilization for musculoskeletal indications in elderly patients with cerebro/cardiovascular disease

**DOI:** 10.1007/s00228-018-2411-y

**Published:** 2018-01-30

**Authors:** Giuseppe Roberto, Claudia Bartolini, Federico Rea, Graziano Onder, Cristiana Vitale, Gianluca Trifirò, Ursula Kirchmayer, Alessandro Chinellato, Ersilia Lucenteforte, Giovanni Corrao, Alessandro Mugelli, Francesco Lapi, Rosa Gini, on behalf of the Italian Group for Appropriate Drug prescription in the Elderly (I-GrADE), Nera Agabiti, Claudia Bartolini, Roberto Bernabei, Alessandra Bettiol, Stefano Bonassi, Achille Patrizio Caputi, Silvia Cascini, Alessandro Chinellato, Giovanni Corrao, Marina Davoli, Massimo Fini, Rosa Gini, Francesco Giorgianni, Ursula Kirchmayer, Francesco Lapi, Niccolò Lombardi, Ersilia Lucenteforte, Alessandro Mugelli, Graziano Onder, Federico Rea, Giuseppe Roberto, Chiara Sorge, Michele Tari, Gianluca Trifirò, Alfredo Vannacci, Davide Liborio Vetrano, Cristiana Vitale

**Affiliations:** 1 Agenzia Regionale di Sanità della Toscana, Via Pietro Dazzi 1, 50141 Florence, Italy; 2 Università degli studi di Milano-Bicocca, Milan, Italy; 3 Università Cattolica Sacro Cuore, Rome, Italy; 4 IRCCS San Raffaele Pisana, Rome, Italy; 5 Università degli studi di Messina, Messina, Italy; 6 Dipartimento di Epidemiologia, ASL Roma 1, Rome, Italy; 7 ULSS 9 Treviso, Treviso, Italy; 8 Università degli studi di Firenze, Florence, Italy

**Keywords:** NSAIDs, Drug utilization, Elderly, Cardiovascular risk, Diclofenac, Coxibs

## Abstract

**Objectives:**

To describe NSAID utilization for musculoskeletal conditions in a large cohort of Italian elderly with cerebro/cardiovascular disease, a population in which NSAIDs should be generally avoided due to the prothrombotic potential.

**Methods:**

Administrative data from five Italian geographic areas were analyzed. Patients aged ≥ 65 with a cerebro/cardiovascular event recorded between 2008 and 2011 (cohort entry) were selected. Prescription NSAIDs reimbursed for musculoskeletal conditions and dispensed during 1 year follow-up were retrieved to describe (i) prevalence of use, (ii) average amount of defined daily doses of NSAIDs claimed by users per day of follow-up, and (iii) distribution of the received daily dose (RDD) among patients with ≥ 2 dispensings. Among new users, i.e., patients without NSAID dispensings during 2 years before cohort entry, the first dispensed NSAID molecule was observed.

**Results:**

Overall, 511,989 patients were selected. Across the five geographic areas, prevalence of use ranged from 48 to 21% and average consumption ranged between 30 and 67 DDD/1000 users/day. Around 10% of patients in the overall cohort had a RDD > 1. Nimesulide (9.6%) and diclofenac (7.5%) had the highest prevalence of use. The most consumed NSAIDs were nimesulide and coxibs with 10.6 and 7.5 DDD/1000 users/day, respectively. Among new users recruited in 2011, 30% had diclofenac or a coxibs as the first prescription.

**Conclusions:**

NSAID use was common in the study cohort, particularly in central-southern areas. In contrast with current recommendations, coxibs and diclofenac were among the most prescribed active principles, even in new users. Interventions to promote appropriateness of use are warranted.

**Electronic supplementary material:**

The online version of this article (10.1007/s00228-018-2411-y) contains supplementary material, which is available to authorized users.

## Introduction

Non-steroidal anti-inflammatory drugs (NSAIDs) are widely used in clinical practice for the symptomatic treatment of common medical conditions causing pain, fever, and inflammation [1].

The analgesic, antipyretic, and anti-inflammatory effects of NSAIDs are due to the inhibition of the cyclooxygenase enzyme (COX) which catalyzes the synthesis of prostanoids, including thromboxane and prostaglandins, and mediates different biological effects [2].

Although all NSAIDs have comparable therapeutic efficacy, they may have different safety profiles in terms of gastrointestinal, cardiovascular, renal, or liver toxicity [3]. As concerns the cardiovascular safety, all non-aspirin NSAIDs are likely to increase the risk of adverse cardiovascular events, though some specific active principles are considered more hazardous than others [2–5]. Available NSAIDs are commonly distinguished in two main categories, “traditional” NSAIDs, generally regarded as non-selective with respect to the inhibition of the two main isoforms of the cyclooxygenase enzyme COX-1/COX-2, and “coxibs”, which show a pronounced selectivity towards COX-2 blockage. Notably, although the hypothesis of COX-2 selectivity explaining the NSAID-related prothrombotic risk has been challenged and, by some, discarded [6], consolidated evidence suggests that the use of coxibs, but also diclofenac, a traditional NSAID with high selectivity for COX-2, can significantly increase the risk of major cerebro/cardiovascular (CCV) events, especially when used at high doses and/or for long periods of treatment [2, 4, 5, 7]. A recent meta-analysis of randomized trials reported a relative risk (RR) of 1.37, (95% confidence interval [CI] 1.14–1.66) for coxibs and 1.41 (95%CI 1.12–1.78) for diclofenac, versus placebo [4]. Comparable results were also reported from a large meta-analysis of observational studies [5]. Moreover, the latter study also showed that baseline cardiovascular risk did not seem to modify the relative risk of adverse cardiovascular events associated to NSAID use compared to no use [4, 5, 8], although a positive interaction in those with CCV diseases or with older age (e.g., 80+) cannot be excluded [9, 10].

In Italy, over 20 different NSAID molecules are currently on the market [11] and can be purchased either with or without medical prescription depending on the specific active substance, formulation, and indication of use. In particular, the Italian National Healthcare Service (NHS) limits the reimbursement of NSAID-containing medicines to some formulation for systemic use, only when prescribed for specific indications, mostly musculoskeletal conditions (i.e., arthropathies, osteoarthritis, gout) [12].

Current recommendations for the use of NSAIDs for the symptomatic treatment of musculoskeletal indications in patients at high cardiovascular risk [2] limit the administration of these medicines to those cases in which other pharmacotherapies (e.g., paracetamol with/without weak opioids) resulted ineffective. The lowest effective dose for the shortest duration should be used. Moreover, among available active principles, naproxen should be regarded as the safest NSAID in this population, while the use of coxibs or diclofenac is strongly discouraged [2, 4, 5].

Given the wide spread use of NSAIDs, serious cardiovascular events due to possible inappropriate utilization behaviors might become a public health issue, particularly in those populations with high baseline risk [1, 4, 7]. With this respect, elderly patients represent a population in which CCV diseases and severe musculoskeletal conditions frequently coexist [13, 14], so that NSAID use is likely to remain in many cases necessary in spite of general contraindications.

In this context, evidence from drug utilization studies becomes fundamental to identify possible signals of irrational uses, discuss measures and interventions to improve prescribing habits, and estimate the magnitude of potential safety issues [15]. However, little is currently known on the real-world patterns of utilization of NSAIDs in such a special population [2, 14, 16].

Therefore, the aim of this study was to describe the pattern of utilization of prescription NSAIDs reimbursed for the treatment of musculoskeletal conditions in a large cohort of elderly patients with CCV disease from five Italian geographic areas.

## Materials and methods

### Source of data

Italy has a tax-based, universal coverage Italian National Healthcare Service (NHS) organized in three levels: national, regional, and local. Local Health Authorities (LHA) are responsible for managing healthcare services delivered to all subjects registered with a general practitioner in the corresponding geographic areas, while the monitoring and promotion of the appropriate use of healthcare resources, including the issue of drug safety advisories to prescribers and pharmacists, can be planned and implemented either at national, regional, or local level.

The utilization of healthcare services reimbursed by the NHS is recorded in administrative databases which allow linking patients’ demographics to records of prescription drugs dispensed to outpatients, as well as to hospital discharge records. Records of drug dispensing for outpatient use include information on the dispensed medicine (active substance, Anatomical Therapeutic Chemical-ATC code [17], brand name, formulation), the number of boxes, and the date of dispensing, though the indication of use for which the drug is prescribed is not recorded. Hospital discharge records provide information on diagnoses and medical procedures, coded with ICD9CM terminology, as well as the dates relevant to each hospitalization event.

The data used for the present study were retrieved from the administrative databases of five health authorities participating to the I-GrADE project (Italian Group for Appropriate Drug Prescription in the Elderly) that was funded by the Italian Medicines Agency. The databases collect information from five Italian geographic areas, corresponding to three regions, Lazio (Center), Toscana (Center), and Lombardia (North), and two Local Health Authorities, Caserta (South) and Treviso (North). The catchment area of these databases covers about 20 million inhabitants, corresponding to over 30% of the Italian population [18].

### Cohort selection and study design

This was a multi-database, descriptive, population-based, retrospective cohort study. Administrative data collected in the five above mentioned geographic areas between 2008 and 2012 were used. All patients with a hospital diagnosis of CCV disease (Appendix 1 - electronic supplementary material) recorded between January 1, 2008 and December 31, 2011 were selected (cohort entry), except for the Lazio region where the latest date of cohort entry was June 30, 2010 due to limited data availability. Patients younger than 65 years at cohort entry or with less than 2 years of look-back period were excluded. Moreover, patients diagnosed with cancer at any time prior to the entry date were excluded.

All patients in the study cohort were followed-up for 1 year after cohort entry or until the occurrence of one of the following events, whichever came first: acute CCV event (Appendix 2 - electronic supplementary material), cancer, death, end of the study period (December 31, 2012).

For each patient in the cohort, all reimbursed dispensings of systemic NSAIDs (ATC code: M01A*) recorded up to 1 year after cohort entry were retrieved and analyzed. Although indication of drug use is not usually recorded in the administrative data sources used for this study, systemic NSAID-containing medicines can be reimbursed by the Italian NHS if prescribed for the treatment of musculoskeletal conditions (i.e., arthropathies, osteoarthritis, gout) and neoplasm-related pain [12]. Therefore, by excluding cancer patients from the study cohort, we reasonably assumed that the observed NSAID dispensings were actually used for the treatment of musculoskeletal conditions.

### Statistical analysis

The characteristics of the selected patients, both in the overall study population and per geographic area, were described in terms of age, gender, comorbidities, and concomitant pharmacotherapies (Appendixes 3 and 4 - electronic supplementary material). Continuous variables were described as mean values; categorical variables as percentages.

Trends of the prevalence of use (percentage of patients with ≥ 1 dispensings during 1 year after cohort entry) and average daily consumption among prevalent users were observed per year of cohort entry, geographic area, and specific NSAID molecule. The average daily NSAID consumption was measured using the defined daily dose (DDD) [17] and expressed as DDD/1000users/day.

To identify patients that were possibly exposed to high daily doses of NSAIDs (i.e., more than 1 DDD per day) [2], the distribution of the received daily dose (RDD) was investigated among patients with ≥ 2 dispensings of any NSAID during follow-up [19]. Although the actual patient usage of drugs cannot be determined using electronic databases, the RDD provides an approximation of patients’ daily exposure to NSAIDs based on the amount of drug actually claimed by patients between the first and last observed NSAID dispensing. Therefore, the RDD was calculated by applying the following formula: [total DDD between first and last dispensing observed during follow-up]/[days of follow-up between first and last dispensing + the duration of the last dispensing], where the duration of the last dispensing was equal to the number of dispensed DDD.

We also investigated the percentage of new users, i.e., patients without any NSAID dispensing during the 2 years preceding cohort entry and with 1 or more NSAID prescriptions during follow-up. Within this sub-population, also the average daily consumption was described. Moreover, new users were classified on the basis of the first active substances dispensed after cohort entry, and the percentage of new users per active substance and year of cohort entry was reported.

All the analyses were performed using the statistical software STATA, version 13.1.

## Results

The overall study population comprised 511,989 elderly patients with CCV disease. Patients’ characteristics at cohort entry are reported in Table S1 (available in the electronic supplementary material).

Prevalence of NSAID use per geographic area ranged from 20.8% in Treviso to 47.8% in Caserta (Table 1). The prevalence of NSAID use per year in the total study population decreased from 30.7% in 2008 to 23.3% in 2011, with a similar trend of decrease in all the geographic areas considered, except for Lazio. In the overall study cohort, the highest prevalence of use per active substance was observed for nimesulide (9.6%) and diclofenac (7.5%), while 3.8% of patients received at least one dispensing of a coxib and 0.7% received naproxen.

**Table 1:** Prevalence of use of NSAIDs during the first year of observation, according to geographic area

	Caserta	Lazio	Toscana	Lombardia	Treviso	Total
Patients, *N*	19.610	84.351	130.664	265.221	12.163	511.989
Prevalence of use (crude), %	47.8	41.8	29.7	22.8	20.8	28.6
Prevalence of use (age-sex standardized), %	46.6	41.7	30.2	22.7	21.1	28.6
Prevalence of use (age-sex standardized) by year of cohort entry, %						
2008	48.7	41.7	31.6	24.4	22.6	30.7
2009	48.1	41.9	30.6	23.0	21.6	29.8
2010	44.7	41.2	29.7	22.2	20.6	27.5
2011	42.5		28.1	19.8	18.2	23.3
Prevalence of use (crude) by active substance, %^1^						
Nimesulide	20.0	17.9	10.0	6.4	3.1	9.6
Diclofenac	13.4	9.7	10.1	5.1	4.0	7.5
Ketoprofen	16.8	10.8	3.9	3.6	3.2	5.4
Ibuprofen	4.8	5.8	5.8	4.9	6.2	5.3
Coxibs^2^	7.3	7.1	3.1	2.8	3.7	3.8
Ketorolac	6.5	4.4	2.3	1.4	1.9	2.4
Piroxicam	3.7	2.3	1.7	1.7	1.3	1.9
Aceclofenac	2.6	1.8	1.0	1.2	1.2	1.3
Meloxicam	2.0	1.3	1.0	0.6	0.5	0.9
Naproxen	1.3	1.1	0.6	0.6	0.3	0.7

^1^Active substances with values lower than those observed for Naproxen were not reported

^2^The category coxibs contains both celecoxib and etoricoxib

The average daily consumption among prevalent users ranged from 29.9 DDD/1000users/day in Treviso to 67.1 DDD/1000 users/day in Lazio (Table 2). The highest consumption in the total study cohort per active substance was observed for nimesulide and coxibs (10.6 and 7.5 DDD/1000 users/day, respectively) followed by diclofenac (7.2 DDD/1000 users/day). The average daily consumption of naproxen among NSAID users was 1.3 DDD/1000 users/day. Between 2008 and 2011, the NSAID consumption in the study cohort increased from 35.6 to 77.2 DDD/1000 users/day, consistently across the five geographic areas.

**Table 2:** Amount of NSAIDs dispensed to prevalent users during the first year of follow-up, according to geographic area

	Caserta	Lazio	Toscana	Lombardia	Treviso	Total
Prevalent users, *N*	9365	35,268	38,900	60,513	2534	146,580
DDD/1000 users/day (crude)	47.7	67.1	37.3	39.9	29.9	44.4
DDD/1000 users/day (age-sex standardized)	47.5	67.1	37.2	39.8	29.9	44.3
DDD/1000 users/day (age-sex standardized) by year of cohort entry						
2008	36.9	54.3	27.9	30.9	27.9	35.6
2009	46.1	75.8	34.9	37.0	26.8	44.7
2010	55.0	117.0	44.8	50.7	34.3	55.8
2011	97.2	–	73	80.7	40.8	77.2
DDD/1000 users/day (crude), by active substance^1^						
Nimesulide	12.3	18.7	9.8	8.1	2.6	10.6
Coxibs^2^	7.3	13.2	4.7	6.9	7.6	7.5
Diclofenac	6.7	8.2	7.8	6.5	4.9	7.2
Ketoprofen	9.6	10.2	2.9	3.9	3.2	5.2
Ibuprofen	2.3	4.8	5.0	5.6	5.3	5.1
Piroxicam	1.8	2.3	1.4	1.8	1.1	1.8
Aceclofenac	1.6	1.8	0.9	1.7	1.4	1.5
Meloxicam	1.6	1.8	1.3	1.3	0.9	1.4
Naproxen	1.2	1.9	0.9	1.5	0.6	1.3

^1^Active substances with values lower than those observed for naproxen were not reported

^2^The category coxibs contains both celecoxib and etoricoxib

**Fig. 1:**
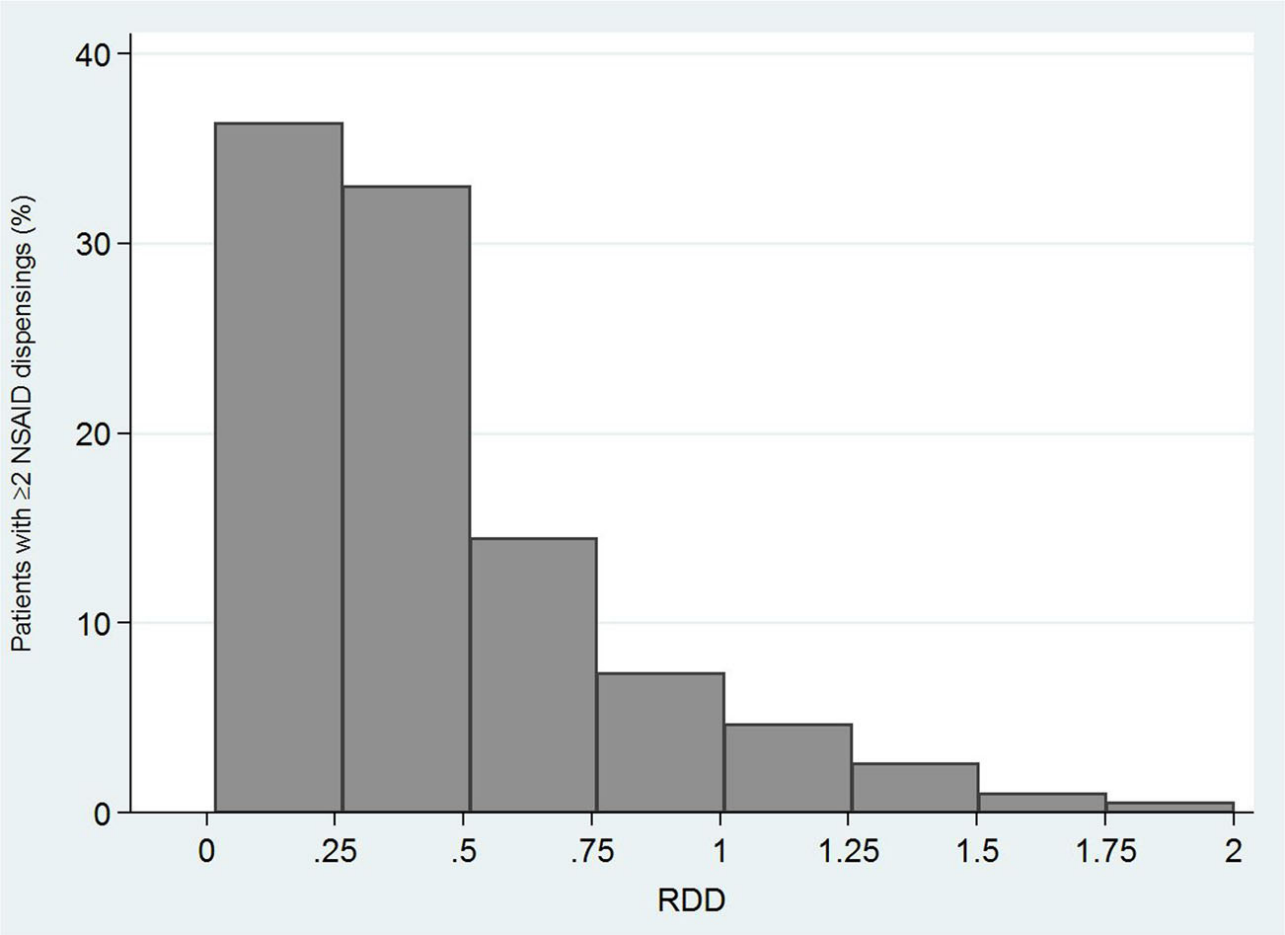
Distribution of the received daily dose among patients with at least two NSAIDs dispensings during follow-up. RDD received daily dose

Around 10% of prevalent users with at least two dispensings during follow-up had a RDD > 1 (Fig. 1). No major differences with respect to the distribution of the RDD were observed across years of cohort entry or geographic areas (data not shown).

**Fig. 2:**
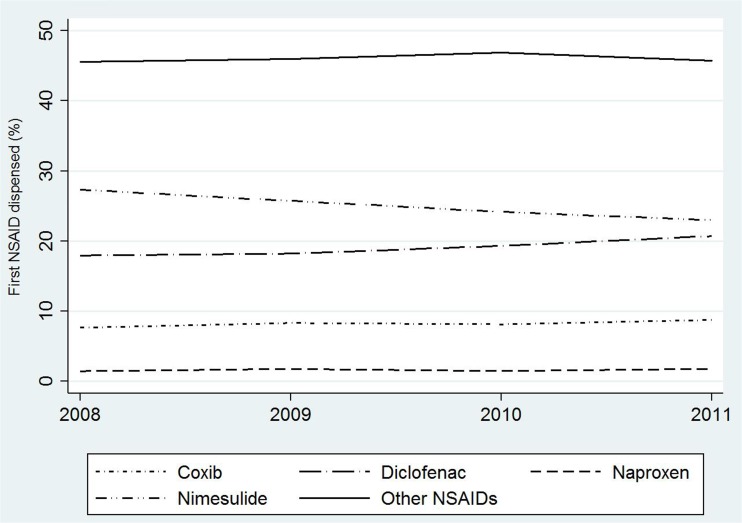
Percentage of new users of NSAIDs according to first dispensed molecule and year of cohort entry

New users accounted for 6.2% of the overall study cohort (Table S2 - electronic supplementary material). The average daily consumption was 23.7 DDD/1000 users/day (Table S3 - electronic supplementary material). In each year of the study period, around 2% of new users received naproxen as the first dispensed molecule (Fig. 2). Coxibs were dispensed in around 8% of new users that entered the study in 2008 and in 9% of those recruited during 2011. The percentage of patients who received diclofenac as the first dispensed NSAID ranged between 18% among new users recruited in 2008 and 21% in 2011.

## Discussion

Results from this study provided evidence on the real-world utilization of NSAIDs for musculoskeletal conditions in a large cohort of elderly with CCV disease from five Italian geographic areas. Overall, the prevalence of use was about 30%, although a remarkable variability across the different areas considered was observed. Average daily NSAID consumption among prevalent users was also inconsistent across geographical areas. In particular, the observed values of these two measures of drug utilization were about two-fold higher in Caserta and Lazio (central-South Italy) compared to Lombardia and Treviso (North). Such variability observed is unlikely to be completely explained by the known higher tendency to the private drug purchasing in northern Italian regions [20] and/or by the underlying differences in frequency of musculoskeletal conditions (prevalence of arthritis/osteoarthritis estimated from national survey data is 13.6% in Lombardia and 18% in Campania) [13]. Rather, these findings might reflect an excessive and inappropriate use of NSAIDs in patients at high CCV risk in the areas of central and South Italy compared to the North.

Overall trends of NSAID utilization during the study period showed a decrease of the prevalence of use over time, with a concomitant increase of the average daily consumption per NSAID user. Probably, the increasing awareness on the potential CCV toxicity of NSAIDs led clinicians to avoid prescribing these medicines to those patients for whom NSAID use was not strictly necessary and for which safer alternatives were available [2].

The distribution of the RDD showed that most of NSAID users with ≥ 2 dispensings during follow-up were treated with low doses and/or for short treatment periods. However, one out of ten patients were possibly exposed to high doses of NSAID (i.e., > 1 DDD per day of treatment).

The utilization of the specific active substances observed in the present study cohort showed a clear disagreement with current knowledge and recommendations on NSAIDs use [2, 4, 5, 21]. Nimesulide, a traditional NSAID with a high selectivity for the COX-2 [2, 22], was by far the most prescribed active principle even though available evidence on cardiovascular safety cannot be considered conclusive [3, 23–25]. Notably, due to concerns on liver toxicity, the indications of use of nimesulide have been progressively restricted: in 2007, treatment duration was limited to a maximum of 15 days; in 2010, the drug was no longer indicated as first-line pharmacotherapy; and, finally, in 2012, nimesulide use was further restricted to the symptomatic treatment of acute pain, excluding painful osteoarthritis [26]. As a consequence of such regulatory interventions, a slight decrease of the prescription of nimesulide in new NSAID users was observed. Diclofenac and coxibs were among the most prescribed NSAIDs, following nimesulide. On the basis of consolidated evidence, these active substances are currently contraindicated in patients with, or at high risk for, cardiovascular disease [2, 7, 27]. Conversely, naproxen that is considered the safest NSAID in this population [2, 4, 5] was strongly underutilized. Notably, as for coxibs, regulatory actions were already undertaken in 2005 to limit their use in patients at risk for CCV events [27]. However, no impact on the prescription pattern of coxib-containing medicines was observed in the present study cohort of elderly patients with CCV disease. The utilization of selective COX-2 inhibitors was high, even among new users, and the percentage of those receiving a coxib as the first NSAID dispensing did not change during the study period.

The main strength of this study is represented by the large scale of the study cohort. Moreover, to the best of our knowledge, this is the first study focusing on NSAID utilization for musculoskeletal conditions in a large cohort of elderly patients with CCV disease. The possibility of comparing NSAID utilization across different geographical areas of the Italian territory represents a further strength of this study, since it facilitated the contextualization and critical discussion of findings. As concerns study limitations, first, the actual NSAID exposure in the study cohort might have been slightly underestimated since non-reimbursed NSAIDs, including both prescription and over-the-counter formulations, are not captured in the data source used for this study. Nevertheless, exposure misclassification in the present cohort is likely to be minor since elderly patients have frequent encounters with physicians, and NSAIDs are fully reimbursed when prescribed for the treatment of musculoskeletal conditions. Second, the indication of the use of the reimbursed NSAID dispensings observed in this study was not validated. However, among all the observed NSAID dispensings, only few injectable formulations could be reimbursed regardless of indication, though those approved for other conditions (e.g., postoperative pain), accounted for less than 10% of all observed dispensings in our study cohort.

In conclusion, results of this study showed that NSAIDs are commonly prescribed for the treatment of musculoskeletal conditions in elderly patients with CCV disease. Comparing the observed patterns of utilization across different geographic areas and with current recommendations on NSAID use, our findings highlighted some inappropriate prescribing behaviors that should be addressed in order to promote a safer use of these medicines. With this respect, a possible overutilization was observed in the central-southern geographic areas concerned. In particular, an extensive prescription of nimesulide, coxibs, and diclofenac was highlighted, even in new NSAID users. Considering the widespread use of NSAIDs worldwide, CCV events potentially attributable to the inappropriate use of these drugs should be considered a public health issue.

## Electronic supplementary material

ESM 1 (DOC 162 kb)
