# Supplementary material for: NSAIDs utilization for musculoskeletal indications in elderly patients with cerebro/cardiovascular disease
Source: Eur J Clin Pharmacol. 2018 Jan 30;74(5):637–43. doi: 10.1007/s00228-018-2411-y (PMC5893698; doi:10.1007/s00228-018-2411-y)
Supplement: Supplementary file 1 — (DOC 162 kb) [file 228_2018_2411_MOESM1_ESM.doc]

**Supplementary material**

**Table S1. Characteristics of patients included in the study cohort.**

|  | Caserta | Lazio^1^ | Toscana | Lombardia | Treviso | Total |
| --- | --- | --- | --- | --- | --- | --- |
| N | 19610 | 84351 | 130644 | 265221 | 12163 | 511989 |
| Women, % | 52.8 | 51.2 | 53.3 | 52.5 | 53.6 | 52.5 |
| Age classes, % |  |  |  |  |  |  |
| 65-74 | 40.2 | 32.0 | 28.1 | 34.3 | 29.7 | 32.4 |
| 75-84 | 43.9 | 45.0 | 42.7 | 42.9 | 41.7 | 43.2 |
| 85+ | 15.8 | 22.9 | 29.3 | 22.8 | 28.6 | 24.4 |
| Year of cohort entry, % |  |  |  |  |  |  |
| 2008 | 33.1 | 48.3 | 32.4 | 33.9 | 34.2 | 35.8 |
| 2009 | 27.1 | 35.3 | 24.9 | 25.2 | 25.8 | 26.8 |
| 2010 | 22.8 | 16.4 | 22.7 | 22.1 | 21.8 | 21.4 |
| 2011 | 17.0 | . | 19.9 | 18.8 | 18.2 | 15.9 |
| CCV disease before cohort entry^2^, % | 34.2 | 26.8 | 46.6 | 44.7 | 24.3 | 41.3 |
| Comorbidities^3^, % |  |  |  |  |  |  |
| Hypertension | 83.1 | 82.0 | 80.3 | 80.1 | 78.8 | 80.5 |
| Dyslipidemia | 30.5 | 32.3 | 25.7 | 28.9 | 28.6 | 28.7 |
| Diabetes | 28.5 | 24.4 | 20.7 | 20.6 | 19.1 | 21.5 |
| COPD | 5.9 | 3.3 | 5.0 | 5.1 | 2.6 | 4.7 |
| Renal disease | 1.6 | 1.6 | 2.3 | 2.3 | 1.0 | 2.1 |
| Gastrointestinal disease | 2.5 | 0.8 | 2.0 | 1.7 | 0.5 | 1.6 |
| Tyroid disease | 1.1 | 0.6 | 1.3 | 0.8 | 0.2 | 0.8 |
| Pharmacotherapies^5^,% |  |  |  |  |  |  |
| Low dose aspirin | 47.3 | 41.2 | 43.8 | 37.7 | 39.2 | 40.2 |
| Systemic antibacterial | 69.4 | 55.8 | 58.1 | 47.3 | 37.4 | 52.1 |
| Proton pump inhibitors | 45.8 | 50.2 | 41.3 | 35.1 | 39.3 | 39.6 |
| Antithrombotics | 29.1 | 32.9 | 27.1 | 30.9 | 28.6 | 30.1 |
| Systemic corticosteroid | 18.5 | 11.4 | 20.1 | 7.3 | 7.3 | 11.7 |
| Antidepressant | 13.5 | 13.6 | 22.8 | 14.5 | 10.1 | 16.3 |
| Paracetamol/codeine | 2.2 | 1.8 | 11.8 | 2.7 | 1.5 | 4.8 |
| Paracetamol | 0.1 | 0.3 | 0.1 | 1.1 | 1.2 | 0.7 |

CCV: cerebro/cardiovascular.

COPD: chronic obstructive pulmonary disease.

Patients recruitment from Lazio ended on June 30, 2010.

^2^acute and chronic CCV events recorded during all the available look-back period before cohort entry (**Appendix 1**).

^3^Comorbidities were measured during two years before cohort entry (**Appendix 3)**.

^4^Pharmacotherapies were measured during one year before cohort entry (**Appendix 4**).

**Table S2. Percentage of new users of NSAIDs according geographic area and active substances**

|  | Caserta | Lazio | Toscana | Lombardia | Treviso | Total |
| --- | --- | --- | --- | --- | --- | --- |
| Patients, *N* | 19610 | 84351 | 130644 | 265221 | 12163 | 511989 |
| Percentage of new users (crude) | 5.5 | 6.4 | 6.7 | 5.9 | 5.6 | 6.2 |
| Percentage of new users (age, sex standardized) | 5.4 | 6.4 | 6.7 | 5.9 | 5.6 | 6.2 |
| Percentage of new users (crude) by active substance^2^ |  |  |  |  |  |  |
| Nimesulide | 1.6 | 1.8 | 1.7 | 1.5 | 0.6 | 1.6 |
| Diclofenac | 0.8 | 0.9 | 1.7 | 1.0 | 0.9 | 1.1 |
| Ketoprofen | 0.8 | 1.0 | 0.5 | 0.7 | 0.6 | 0.7 |
| Ibuprofen | 0.2 | 0.6 | 0.6 | 0.4 | 1.4 | 0.5 |
| Piroxicam | 0.4 | 0.2 | 0.4 | 0.6 | 0.2 | 0.5 |
| *Coxibs*^3^ | 0.5 | 0.7 | 0.5 | 0.4 | 0.6 | 0.5 |
| Ketorolac | 0.4 | 0.4 | 0.3 | 0.3 | 0.4 | 0.3 |
| Meloxicam | 0.1 | 0.1 | 0.2 | 0.2 | 0.1 | 0.2 |
| Aceclofenac | 0.2 | 0.1 | 0.2 | 0.1 | 0.2 | 0.1 |
| Naproxene | 0.0 | 0.1 | 0.1 | 0.1 | 0.0 | 0.1 |

Patients without any NSAID dispensing during two years before cohort entry.

^2^ Active substances with values lower than those observed for naproxen were not reported.

^3^ The category *coxibs* contains both celecoxib and etoricoxib.

**Table S3. Amount of NSAIDs dispensed to new users during follow-up, according geographic area and active substances.**

|  | Caserta | Lazio | Toscana | Lombardia | Treviso | Total |
| --- | --- | --- | --- | --- | --- | --- |
| New users, *N* | 1074 | 5432 | 8736 | 15744 | 683 | 31696 |
| DDD/1000 users/day (crude) | 24.1 | 35.5 | 22.0 | 22.3 | 15.9 | 23.7 |
| DDD/1000 users/day (age-sex standardized) | 24.1 | 35.4 | 21.8 | 22.1 | 15.7 | 23.6 |
| DDD/1000 users/day (crude) by active substance^2^ |  |  |  |  |  |  |
| Nimesulide | 5.6 | 8.7 | 4.8 | 3.8 | 1.1 | 4.6 |
| *Coxibs*^3^ | 3.3 | 6.4 | 3.0 | 3.8 | 3.3 | 3.8 |
| Diclofenac | 3.4 | 4.1 | 4.4 | 3.4 | 2.5 | 3.7 |
| Ibuprofene | 2.1 | 3.9 | 2.4 | 2.4 | 2.2 | 3.7 |
| Ketoprofene | 4.6 | 6.2 | 1.9 | 2.4 | 1.7 | 2.8 |
| Piroxicam | 1.0 | 1.0 | 0.9 | 1.0 | 0.7 | 1.0 |
| Aceclofenac | 0.8 | 1.0 | 0.5 | 0.9 | 1.1 | 0.8 |
| Naproxene | 0.9 | 1.0 | 0.5 | 0.8 | 0.2 | 0.7 |

Patients without any NSAID dispensing during two years before cohort entry.

^2^ Active substances with values lower than those observed for naproxen were not reported.

^3^ The category *coxibs* contains both celecoxib and etoricoxib.

**Appendix 1. *Definition of events for cohort entry***

1. ISCHEMIC HEART DISEASE

The selection algorithm for cohort definition includes the following ICD9CM codes in either primary or secondary diagnosis fields:

410.* Acute myocardial infarction

411.* Other acute and subacute forms of ischemic heart disease

412.* Old myocardial infarction

413.* Angina pectoris

414.* Other forms of chronic ischemic heart disease

It will be possible to distinguish different sub-populations:

1a. Acute Myocardial Infarction (AMI)

Primary diagnosis = 410.* OR

Primary diagnosis = codes compatible** with a diagnosis of acute myocardial infarction, including procedure-related complications, and secondary diagnosis = 410.*

**Codes compatible with a diagnosis of acute myocardial infarction

411.* Other acute and subacute forms of ischemic heart disease

413.* Angina pectoris

414.* Other forms of chronic ischemic heart disease

423.0 Hemopericardium

426.* Conduction disorders

429.5 Rupture of chordae tendinae

429.6 Rupture of papillary muscle

429.71 Acquired cardiac septal defect

429.79 Other sequelae of myocardial infarction, not elsewhere classified (Mural thrombus (atrial, ventricular) acquired, following myocardial infarction)

429.81 Other disorders of papillary muscle

518.4 Acute edema of lung, unspecified

518.81 Acute respiratory failure

780.01 Coma

780.2 Syncope and collapse

785.51 Cardiogenic shock

799.1 Respiratory arrest

997.02 Iatrogenic cerebrovascular infarction or hemorrhage (postoperative stroke)

998.2 Accidental puncture or laceration during a procedure (accidental perforation by catheter)

1b. Subacute and chronic forms of ischemic heart disease

Subacute forms and angina pectoris

411.* Other acute and subacute forms of ischemic heart disease

413.* Angina pectoris

Chronic forms

412.* Old myocardial infarction

414.* Other forms of chronic ischemic heart disease

The following codes identify chronic forms when associated with codes 410.* or 414.* in secondary diagnosis fields:

4^2^9.2 Cardiovascular disease unspecified

429.3 Cardiomegaly

429.4 Functional disturbances following cardiac surgery

429.89 Other ill-defined heart diseases

2. HEART FAILURE

The case selection algorithm includes the following ICD9CM codes in either primary or secondary diagnosis fields excluding admissions to rehabilitations wards:

428.* Heart failure

398.91 Rheumatic heart failure (congestive)

402.01 Malignant hypertensive heart disease with heart failure

402.11 Benign hypertensive heart disease with heart failure

402.91 Unspecified hypertensive heart disease with heart failure

404.01 Malignant hypertensive heart and renal disease with heart failure

404.03 Malignant hypertensive heart and renal disease with heart failure and renal failure

404.11 Benign hypertensive heart and renal disease with heart failure

404.13 Benign hypertensive heart and renal disease with heart failure and renal failure

404.91 Unspecified hypertensive heart and renal disease with heart failure

404.93 Unspecified hypertensive heart and renal disease with heart failure and renal failure

The following codes identify heart failure when associated with code 428.* in secondary diagnosis fields:

425.4 Other primary cardiomyopathies

425.5 Alcoholic cardiomyopathy

425.7 Nutritional and metabolic cardiomyopathy

425.8 Cardiomyopathy in other diseases classified elsewhere

425.9 Secondary cardiomyopathy unspecified

3. CARDIAC ARRHYTHMIA

The case selection algorithm includes the following ICD9CM codes in either primary or secondary diagnosis fields:

427.* Cardiac dysrhythmias

785.0 Tachycardia unspecified

4. STROKE

4a. Acute conditions

The case selection algorithm includes the following ICD9CM codes in primary diagnosis fields:

Haemorrhagic Stroke

430 Subarachnoid haemorrhage

431 Intracerebral haemorrhage

432.* Other and unspecified intracranial hemorrhage

Ischemic Stroke

433.* Occlusion and stenosis of precerebral arteries

434.* Occlusion of cerebral arteries

436 Acute but ill-defined cerebrovascular disease

Transient cerebral ischemia

435.* Transient cerebral ischemia

4b. Subacute, chronic and late effects of cerebrovascular disease

The case selection algorithm includes the following ICD9CM codes in primary and secondary diagnosis fields:

437.0 Cerebral atherosclerosis

437.1 Other generalized ischemic cerebrovascular disease

438.* Late effects of cerebrovascular disease

**Appendix 2*. Definition of acute carebro/cardiovascular events****

The selection algorithm for case definition includes the following ICD9CM codes in primary diagnosis field excluding admissions to rehabilitations wards:

A. Cardiovascular events

410.* OR Primary diagnosis = codes compatible* with a diagnosis of acute myocardial infarction, including procedure-related complications, and secondary diagnosis= 410.*

Codes compatible with a diagnosis of acute myocardial infarction

411.* Other acute and subacute forms of ischemic heart disease

413.* Angina pectoris

414.* Other forms of chronic ischemic heart disease

423.0 Hemopericardium

426.* Conduction disorders

427.* Cardiac dysrhythmias (excluding 427.5 Cardiac arrest)

428.* Heart failure

429.5 Rupture of chordae tendineae

429.6 Rupture of papillary muscle

429.71 Acquired cardiac septal defect

429.79 Other sequelae of myocardial infarction, not elsewhere classified (Mural thrombus (atrial) (ventricular) acquired, following myocardial infarction)

429.81 Other disorders of papillary muscle

518.4 Acute edema of lung, unspecified

518.81 Acute respiratory failure

780.01 Coma

780.2 Syncope and collapse

785.51 Cardiogenic shock

799.1 Respiratory arrest

997.02 Iatrogenic cerebrovascular infarction or hemorrhage (postoperative stroke)

998.2 Accidental puncture or laceration during a procedure (accidental perforation by catheter)

B. Cerebrovascular events

Hemorrhagic Stroke

430 Subarachnoid hemorrhage

431 Intracerebral hemorrhage

432.* Other and unspecified intracranial hemorrhage

Ischemic Stroke

433.*1 Occlusion and stenosis of precerebral arteries with cerebral infarction

434.* Occlusion of cerebral arteries

Transient cerebral ischemia

435.* Transient cerebral ischemia

C. Heart failure

428.* Heart failure

402.01 Malignant hypertensive heart disease with heart failure

402.11 Benign hypertensive heart disease with heart failure

402.91 Unspecified hypertensive heart disease with heart failure

404.01 Malignant hypertensive heart and renal disease with heart failure

404.03 Malignant hypertensive heart and renal disease with heart failure and renal failure

404.11 Benign hypertensive heart and renal disease with heart failure

404.13 Benign hypertensive heart and renal disease with heart failure and renal failure

404.91 Unspecified hypertensive heart and renal disease with heart failure

404.93 Unspecified hypertensive heart and renal disease with heart failure and renal failure

*The definition of “*major cerebro/cardiovascular events*” did not include heart failure (C).

**Appendix 3. *Definition of baseline comorbidities***

Hospitalization at cohort entry (secondary diagnosis) or during two years before Index admission (primary and secondary diagnosis).

Diseases and ICD9CM codes

DIABETES

250.* Diabetes mellitus

OR

ATC code A10* - Drugs used in diabetes

COPD

491.* Chronic bronchitis

492.* Emphysema

493.* Asthma

496 Chronic airway obstruction not elsewhere classified

OR

Primary diagnosis = codes compatible** with a diagnosis of COPD and secondary diagnosis = 491.*, 492.*, 493.*, 496

**Codes compatible with diagnosis of COPD

518.81 Acute respiratory failure

518.83 Chronic respiratory failure

518.84 Acute and chronic respiratory failure

416.8 Other chronic pulmonary heart diseases

416.9 Chronic pulmonary heart disease unspecified

GASTROINTESTINAL DISEASES

530.11 Reflux esophagitis

530.81 Esophageal reflux

531.4* Chronic or unspecified gastric ulcer with hemorrhage

531.5* Chronic or unspecified gastric ulcer with perforation

531.6* Chronic or unspecified gastric ulcer with hemorrhage and perforation

531.7* Chronic gastric ulcer without mention of hemorrhage or perforation

531.9* Gastric ulcer unspecified as acute or chronic without mention of hemorrhage or perforation

532.4* Chronic or unspecified duodenal ulcer with hemorrhage

532.5* Chronic or unspecified duodenal ulcer with perforation

532.6* Chronic or unspecified duodenal ulcer with hemorrhage and perforation

532.7* Chronic duodenal ulcer without mention of hemorrhage or perforation

532.9* Duodenal ulcer unspecified as acute or chronic without mention of hemorrhage or perforation

533.4* Chronic or unspecified peptic ulcer of unspecified site with hemorrhage

533.5* Chronic or unspecified peptic ulcer of unspecified site with perforation

533.6* Chronic or unspecified peptic ulcer of unspecified site with hemorrhage and perforation

533.7* Chronic peptic ulcer of unspecified site without mention of hemorrhage or perforation

533.9* Peptic ulcer of unspecified site unspecified as acute or chronic without mention of hemorrhage or perforation

534.4* Chronic or unspecified gastrojejunal ulcer with hemorrhage

534.5* Chronic or unspecified gastrojejunal ulcer with perforation

534.6* Chronic or unspecified gastrojejunal ulcer with hemorrhage and perforation

534.7* Chronic gastrojejunal ulcer without mention of hemorrhage or perforation

534.9* Gastrojejunal ulcer unspecified as acute or chronic without mention of hemorrhage or perforation

535* Gastritis and duodenitis

553.3 Diaphragmatic hernia without obstruction or gangrene

DISORDERS OF THYROID GLAND

240* Simple and unspecified goiter

241* Nontoxic nodular goiter

242* Thyrotoxicosis with or without goiter

243* Congenital hypothyroidism

244* Acquired hypothyroidism

245* Thyroiditis

246* Other disorders of thyroid

HYPERTENSION

401.* Essential hypertension

OR ATC C02*, C03*, C07*, C08*, C09* (at least two prescription before cohort entry)

HYPERCHOLESTEROLEMIA

272.0 Pure hypercholesterolemia

HYPERTRIGLYCERIDEMIA

272.1 Pure hyperglyceridemia

DYSLIPIDEMIA

272.2 Mixed hyperlipidemia

OR ATC: C10* (at least two prescription in the year before the cohort entry)

RENAL FAILURE

584.* Acute renal failure

585.* Chronic kidney disease

OTHER FACTORS RELATED TO SEVERITY OF DISEASE

Cardiovascular procedures

CORONARY ARTERY BYPASS SURGERY

36.1* Bypass anastomosis for heart revascularization

V45.81 Postsurgical aortocoronary bypass status

PTCA

00.66 Percutaneous transluminal coronary angioplasty [ptca] or coronary atherectomy

36.0* Removal of coronary artery obstruction and insertion of stent(s)

V45.82 Percutaneous transluminal coronary angioplasty status

CEREBRAL REVASCULARIZATION

00.61 Percutaneous angioplasty or atherectomy of precerebral (extracranial) vessel(s)

00.62 Percutaneous angioplasty or atherectomy of extracranial vessel(s)

38.11 Endarterectomy, intracranial vessels

38.12 Endarterectomy, other vessels of head and neck

38.31 Resection of vessel with anastomosis, intracranial vessels

38.32 Resection of vessel with anastomosis, other vessels of head and neck

OTHER HEART SURGERY PROCEDURES

35.* Operations On Valves And Septa Of Heart

37.0 Pericardiocentesis

37.1* Cardiotomy and pericardiotomy

37.3* Pericardiectomy and excision of lesion of heart

37.4* Repair of heart and pericardium

37.5* Heart replacement procedures

37.6* Implantation of heart assist system

37.9* Other operations on heart and pericardium

**Appendix 4. *Definition of pharmacotherapies***

Antithrombotics (ATC: B01A*, excluding low-dose aspirin),

Low-dose aspirin (ATC: B01AC06, B01AC56)

Proton pump inhibitors (ATC: A02BC*)

Corticosteroids for systemic use (ATC: H02*)

Paracetamol (ATC: N02BE01, N02BE51, N02BE71)

Paracetamol and association with codeine (ATC: N02AA59, N02BE51)

Antibacterial for systemic use (ATC: J01*)

Antidepressants (ATC: N06A*)

Antipsychotic drugs (ATC: N05A*)

Anti-dementia drugs (ATC: N06D*)
